# Rotavirus infection in children in Southeast Asia 2008–2018: disease burden, genotype distribution, seasonality, and vaccination

**DOI:** 10.1186/s12929-020-00649-8

**Published:** 2020-05-21

**Authors:** Fajar Budi Lestari, Sompong Vongpunsawad, Nasamon Wanlapakorn, Yong Poovorawan

**Affiliations:** 1 Inter-Department of Biomedical Science, Faculty of Graduate School, Chulalongkorn University, Bangkok, Thailand; 2 Department of Bioresources Technology and Veterinary, Vocational College, Universitas Gadjah Mada, Yogyakarta, Indonesia; 3 Center of Excellence in Clinical Virology, Faculty of Medicine, Chulalongkorn University, Bangkok, 10330 Thailand; 4 Division of Academic Affairs, Faculty of Medicine, Chulalongkorn University, Bangkok, Thailand

**Keywords:** Rotavirus, Disease burden, Genotypes, Vaccination, Southeast Asia

## Abstract

**Background:**

Rotaviruses (RVs) are recognized as a major cause of acute gastroenteritis (AGE) in infants and young children worldwide. Here we summarize the virology, disease burden, prevalence, distribution of genotypes and seasonality of RVs, and the current status of RV vaccination in Southeast Asia (Cambodia, Indonesia, Lao People’s Democratic Republic, Malaysia, Myanmar, Philippines, Singapore, Thailand, and Vietnam) from 2008 to 2018.

**Methods:**

Rotavirus infection in Children in Southeast Asia countries was assessed using data from Pubmed and Google Scholars. Most countries in Southeast Asia have not yet introduced national RV vaccination programs. We exclude Brunei Darussalam, and Timor Leste because there were no eligible studies identified during that time.

**Results:**

According to the 2008–2018 RV surveillance data for Southeast Asia, 40.78% of all diarrheal disease in children were caused by RV infection, which is still a major cause of morbidity and mortality in children under 5 years old in Southeast Asia. Mortality was inversely related to socioeconomic status. The most predominant genotype distribution of RV changed from G1P[8] and G2P[4] into the rare and unusual genotypes G3P[8], G8P[8], and G9P[8]. Although the predominat strain has changed, but the seasonality of RV infection remains unchanged. One of the best strategies for decreasing the global burden of the disease is the development and implementation of effective vaccines.

**Conclusions:**

The most predominant genotype distribution of RV was changed time by time. Rotavirus vaccine is highly cost effective in Southeast Asian countries because the ratio between cost per disability-adjusted life years (DALY) averted and gross domestic product (GDP) per capita is less than one. These data are important for healthcare practitioners and officials to make appropriate policies and recommendations about RV vaccination.

## Introduction

### Rotavirus (RV) history

RV was first identified in cattle in 1969 [1]. The virus appeared similar to those that cause diarrhea in mice [2], calves [3], and a virus identified from a rectal swab of a healthy monkey [4]. In May 1973, Bishop, Davidson, Holmes, and Ruck examined ultrathin sections of duodenal mucosa from children with acute gastroenteritis (AGE) by electron microscopy (EM), and found abundant viral particles in the epithelial cell linings of the upper villous surface which were similar in appearance to the RVs discovered in animals before [5]. EM also revealed 70-nm particles in negatively stained fecal extracts [6, 7]. The viral particle was initially identified by several names including reovirus-like, orbivirus-like, duovirus, infantile gastroenteritis virus, or a “new” virus. The wheel-like structure observed on EM eventually led to the naming concensus of Rotavirus (*rota* is Latin for wheel) [8]. RVs have now been shown to be a cause of diarrhea in the young of many mammalian and avian species [9].

### RV morphology

RVs are 70-nm, non-enveloped RNA viruses belonging to the family *Reoviridae*. The RV genome consists of 11 segments of double-stranded RNA (dsRNA) surrounded by a triple-layered capsid. Each genomic fragment encodes protein of different function. The outer layer proteins (viral protein [VP] 4 and VP7) mediate attachment and penetration; the inner layer is composed of VP2 protein and encloses the viral genome and the minor protein VP1, the viral RNA-dependent RNA polymerase, and VP3, the viral capping enzyme. The middle layer is composed of VP6 which interacts with and stabilized the inner and outer layer [9]. VP6 defines species/group and subgroup specificities [10–12]. All RNA segments, except for segment 11, are monocistronic, encoding either structural viral proteins (VP1 to VP4, VP6, and VP7) or non-structural proteins (NSP1 to NSP5). Genome segment 11 codes for two proteins: NSP5 and NSP6 [9]. RVs can be differentiated by a dual classification system, based on the two outer capsid proteins, VP7 and VP4, that determine the G (VP7, glycoprotein) and P (VP4, protease-sensitive) genotypes [13]. At least 36 G types and 51 P types have so far been identified in humans and animals [14].

A whole genome-based genotyping system was recently proposed for RV Group A (RVA) based on the genotype assignment of all 11 gene segments [15]. The genome of individual RV strains is given the complete descriptor of Gx-P [x]-Ix-Rx-Cx-Mx-Ax-Nx-Tx-Ex-Hx to identify the genotypes of the VP7-VP4-VP6-VP1-VP2-VP3-NSP1-NSP2-NSP3-NSP4-NSP5/6 encoding RNA segments, respectively. Most strains demonstrate either a Wa-like (G1-P[8]-I1-R1-C1-M1-A1-N1-T1- E1-H1), DS-1 (G2-P[4]-I2-R2-C2-M2-A2-N2-T2-E2-H2), or AU-1-related (G3-P[3]-I3-R3-C3-M3-A3/A12-N3-T3-E3-H3/H6) genotype constellation [16, 17].

Indirect immunofluorescence techniques targeting VP6 are used to differentiate RV species. RVs are currently differentiated into at least nine species, designated A to I and a tentative tenth species, J. RVA infects in birds and mammals; RVB, RVC, RVE, RVH, and RVI have been detected in one or more mammalian hosts; RVD, RVF, and RVG have been detected only in birds; RVJ infects bats [18–20]. Table 1 shows rotavirus groups and its host.

**Table 1:** Rotavirus groups and hosts

Rotavirus Group	Host
A	Human, Pig [21], Cattle, Horse [22], Rabbit [23], Alpaca [24], Turkey, Pheasant, Bat, Sugar Glider, Camel, Vicugna, Velvet Scoter, Fox, Common Gull, Chicken, Shrew, Racoon, Mouse [1, 16, 25]
	Sheep, Partridge, Panda, Monkey, Mussel, Oyster, Shellfish, Salmon, Shark, Trout, Deer, Mosquito, Cormorant, Fly, Moth, Tick, Tasmanian Devil, Leafhopper, Buffalo, Antelope, Dog, Civet, Cat [26] Giraffe [27] Pigeon, Guanaco, Macaques [28]
B	Human, Cattle, Pig, Rat, Goat [29]
C	Human [30], Dog, Bear, Ferret, Pig [31]
D	Chicken, Duck, Pigeon, Guinea Fowl [25, 32]
E	Pig [33]
F	Pig, Chicken, Teal, Partridge [25, 32]
G	Chicken, Duck, Pigeon, Turkey, Partridge, Gull, Avaret, Teal [25, 32]
H	Human, Pig, Bat [34, 35]
I	Cat [36], Dog [19]
J	Bat [20]

### RV infection burden

RVs were recognized as a major cause of AGE in infants and young children in 1973 [2, 7]. RV is the leading cause of diarrhea-associated mortality among children younger than 5 years, although the burden of RV has decreased during the past decade. RV infections were responsible for approximately 128,515 deaths annually among children younger than 5 years. RV constitutes 1 of the 13 diarrhea etiologic agents measured in the 2016 Global Burden of Disease Study [37]. It is the most prevalent agent causing severe diarrhea in both developed and developing countries [38, 39]. After RV vaccine introduction in developed countries, norovirus become the predominant viral pathogen that caused AGE in children. Norovirus prevalence remained stable or increased, whereas rotavirus activity dramatically decreased [40–42]. Nevertheles, from 2000 to 2013 in Southeast Asia, approximately 50.7% (*n* = 10,765) of total diarrhea mortality was associated with RV disease [43].

**Fig. 1:**
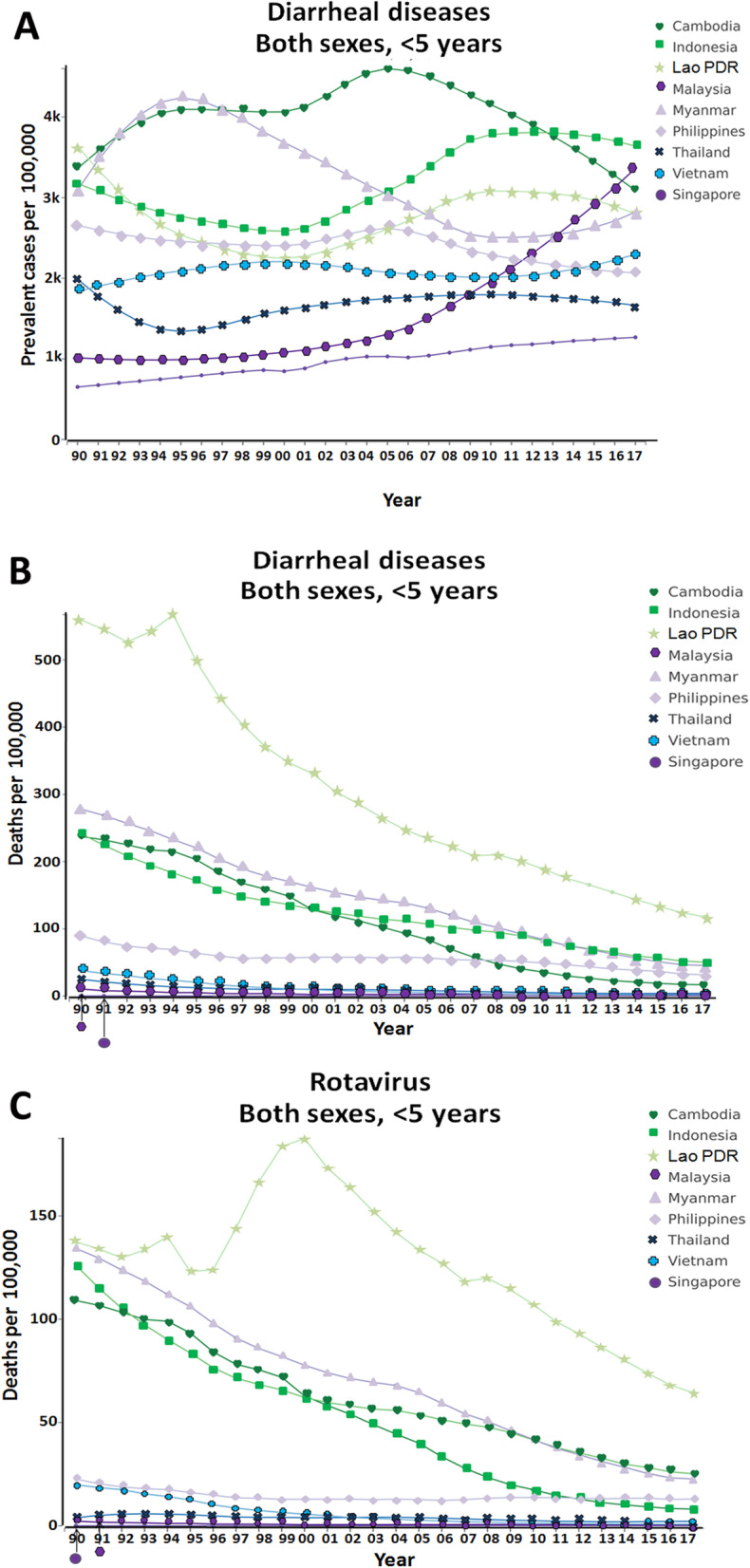
Diarrhea diseases in children under 5 years old in Southeast Asian countries from 1990 to 2017. **a** Prevalence of diarrhea; **b** diarrhea-associated mortality; **c** mortality attributed specifically to rotavirus [44, 45]

Figure 1 shows the prevalence and death caused by diarrheal disease and RV in children under 5 years old from 1990 to 2017 in Southeast Asia. The prevalence of diarrheal diseases in Southeast Asia countries varies, but the mortality trend associated with diarrhea and especially RV infection has been decreasing in recent years. Lao People’s Democratic Republic [PDR] reports one of the highest death rates in this period. However, improvement in hygiene and sanitation combined with the introduction of the rotavirus vaccine has contributed to decreasing RV infection [46].

**Fig. 2:**
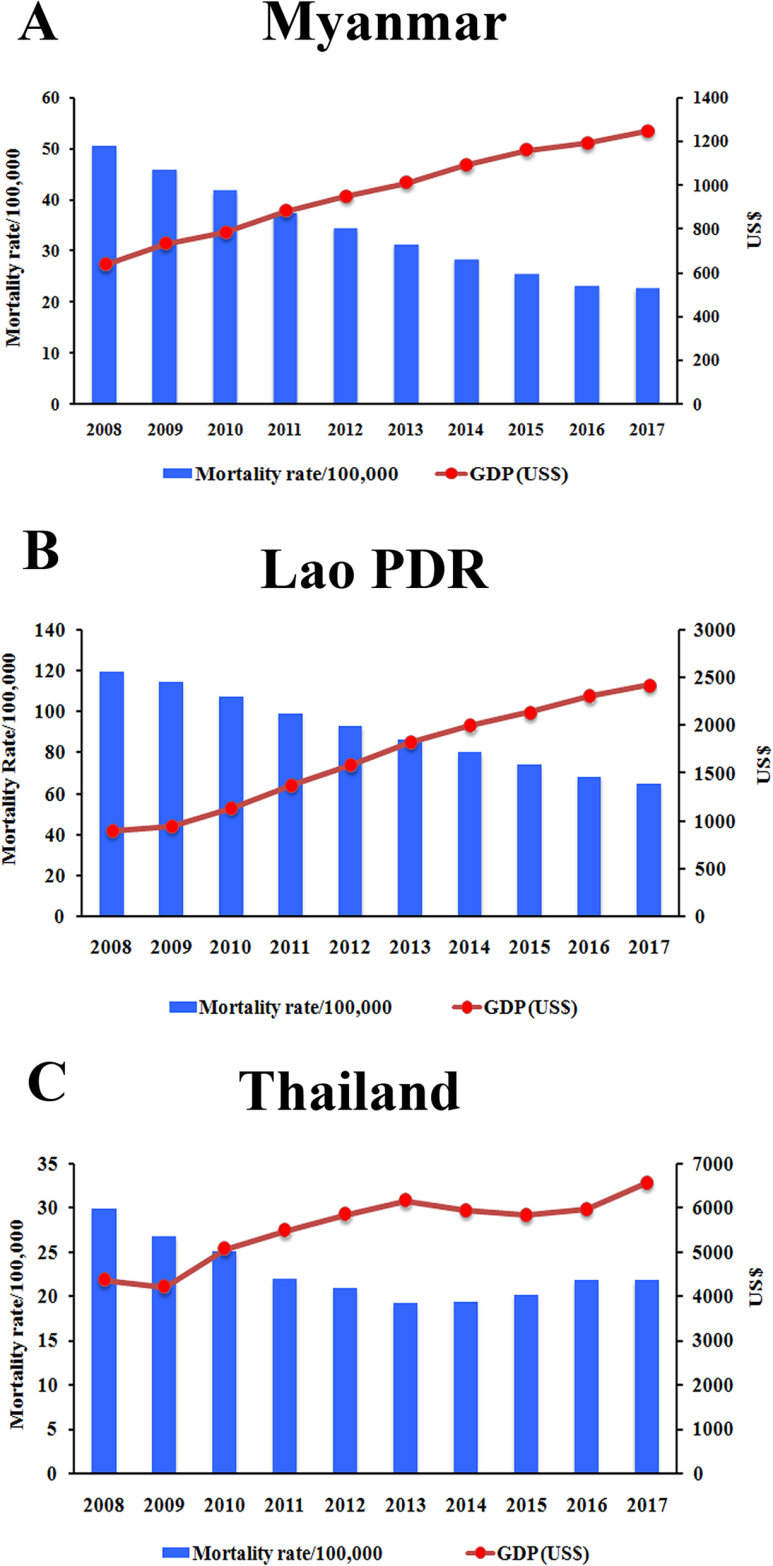
The mortality of rotavirus-associated acute gastroenteritis per 100,000 children under 5 years old and the national gross domestic product (GDP) per capita between 2008 to 2017 in lower-middle income countries; **a** Myanmar and **b** Lao PDR, and in the upper-middle income country; **c** Thailand [44, 79]. The bar graphs represent the mortality rate per 100,000 populations. The red dots represent the GDP per capita in US$

We collected RV surveillance data from 9 countries in Southeast Asia (Cambodia, Indonesia, Lao PDR, Malaysia, Myanmar, Philippines, Singapore, Thailand, and Vietnam) for the years 2008–2018 to estimate the proportion of RV gastroenteritis (RVGE) **(**Table 2**)**. A total of 52,579 stool samples were collected of which 21,444 (40.78%) were RV positive. Acute diarrheal disease caused by RV is still a major cause of morbidity and mortality in children under 5 years old in developing countries, which may be attributed to the regions’ lower standards of living and hygiene conditions [46]. However, our study revealed that as the gross domestic product (GDP) per capita increases, and the economic status of Southeast Asian countries improves, the RV mortality rate steadily declines (Fig. 2). Higher socioeconomic status (SES) can improve sanitation, hygiene practices, and healthcare facilities to support better living conditions and decrease the RV mortality in children.

**Table 2:** The annual incidence of rotavirus in children under 5 years old in Southeast Asian countries, 2007–2018

Country	Region	Year	Study Design	Number of Stool Sample	Number of Rotavirus Positive	Percentage (%)	Reference
Cambodia	Phnom Penh	2010–2016	Active hospital surveillance	7007	3473	49.56	[47]
Indonesia	Bandung, Yogyakarta, Mataram, Denpasar	2009–2010	Hospital-based surveillance	4235	2220	52.42	[48]
	Yogyakarta	2009	Hospital-based surveillance	104	57	54.81	[49]
	Denpasar	2009–2011	Hospital-based surveillance	656	327	49.85	[50]
	Bandung	2009–2012	Prospective cross-sectional study	135	92	68.15	[51]
	Mataram	2010	Cross-sectional study	328	210	64.02	[52]
	Surabaya	2013	Cross-sectional study	220	88	40	[53]
	Pekanbaru	2015	Cross-sectional study	71	42	59.15	[54]
	Indonesia	2010–2015	National surveillance	4013	1950	48.59	[55]
	Surabaya	2015–2016	Hospital-based surveillance	134	42	31.34	[56]
	Central Java	2013–2016	Hospital-based surveillance	1649	105	6.37	[57]
	Jawa Timur	2015–2018	Hospital-based surveillance	432	137	31.71	[58]
Lao PDR	Vientiane	2009–2015	Hospital-based surveillance	1772	928	52.37	[59]
Malaysia	Malaysia	2008–2010	Hospital-based surveillance	822	279	33.94	[60]
Myanmar	Yangon	2009–2014	Prospective active surveillance	3724	1860	49.95	[61]
Philippines	Palawan	2012	Not specified	45	25	55.56	[62]
	Philippines	2013–2015	National Surveillance	5229	2024	38.1	[63]
	Zamboanga city	2016	Hospital-based surveillance	93	56	60.22	[64]
Singapore	Singapore	2008	Hospital-based surveilance	285	167	58.60	[65]
	Singapore	2008	Randomized clinical trial	58	11	18.97	[66]
Thailand	Bangkok, Khon Kaen, Nahon Ratchasima, Tak	2007–2009	Hospital-based surveillance	557	158	28.37	[67]
	Chiang Rai, Nakhon Ratchasima, Surat Thani, Phitsanulok,	2008–2010	Regional surveillance	3470	458	13.20	[68]
	Khon Kaen & Bangkok	2009–2011	Hospital-based surveillance	562	250	44.48	[69]
	Thailand	2010–2013	Active surveillance	1032	184	17.83	[70]
	Khon Kaen, Bangkok	2011–2014	Hospital-based surveillance	688	204	29.65	[71]
	Chiang Mai	2012	Hospital-based surveillance	186	35	18.82	[72]
	Nonthaburi	2012–2014	Hospital-based surveillance	No Data	73	–	[73]
	Sukhothai, Petchabun	2013–2014	Regional surveillance	2754	666	24.18	[74]
	Thailand	2014–2016	Hospital-based surveillance	1867	514	27.53	[75]
	Chiang Rai	2015–2016	Hospital-based surveillance	270	91	33.70	[76]
Vietnam	Ho Chi Min	2009–2010	Hospital-based surveillance	1419	664	46.79	[77]
	Vietnam	2012–2015	National Surveillance	8689	4054	46.66	[78]

### RV genotype distribution

Among the data from Southeast Asia countries examined, the most predominant genotype distribution of RV has changed except in Lao PDR and Malaysia. In 2009–2013, G1P[8] and G2P[4] were the most predominant genotypes, but starting 2014, it changed into the rare and unusual genotypes G3P[8], G8P[8], and G9P[8]. Several uncommon RV genotypes such as G2P[8], G8P[6], G5P[19], G9P[4], G9P[6], and G1P7[5] were identified in the surveillance data. The presence of such diversity among RV isolates provides insight into the evolution of these strains, which can arise due to point mutations, genetic rearrangements, reassortment events, and interspecies transmission [13, 80, 81]. Circulating RV strain appears diverse despite RV vaccination, which may enable the increase in the prevalence of non-vaccine strains. Thus, the circulation of strains in which vaccines have lower efficacy eventually impairs vaccine effectiveness [82]. In the Philippines, where the Rotarix® vaccine was introduced in July 2012, the frequency of RVGE cases caused by G1P[8] decreased while the circulation of G9P[8] increased significantly [83]. The genotype distribution of RV in Southeast Asia is shown in Table 3.

**Table 3:** The distribution of rotavirus genotype in Southeast Asian countries based on the surveillance data from 2008 to 2018^a^

Genotype	Year									
	2008	2009	2010	2011	2012	2013	2014	2015	2016	2017/2018
Cambodia										
G1P[8]			118	109	190	107	52	81	8	
G2P[4]				2	12	199	88			
G3P[8]			21	50	5	4		43	101	
G8P[8]				2	1	4	42	118	8	
G9P[8]			2			4	1	29	29	
Others			19	3	34	52	18	17	14	
Indonesia										
G1P[4]		30	11	16		30				
G1P[6]		33	38	8		33				1
G1P[8]		64	219	98	138	202	2		1	12
G1P[UT]		3		1	3	6				
G2P[4]		35	25	17	1	36	1			
G2P[6]				5	7	7	7	4	5	
G2P[8]			4		3	3				
G3P[4]					1	1				
G3P[6]					1	1		1	4	19
G3P[8]		2			6	8	47	54	126	98
G3P[9]					1	1				
G3P[UT]									7	7
G9P[8]								1		
G12P[8]						8				
Thailand										
G1P[4]		8				4				
G1P[8]	14	217	63	146	87	236	447	42	3	
G2P[4]	38	107		3	5	109	152	3		
G2P[8]	1	11	2			4	1			
G3P[8]	1	22	20	156	26	4	19	125	44	
G3P[9]		1		2			3	2	1	
G4P[6]	1	1	2	1		1	1	1		
G8P[8]			1			58	164	8		
G9P[8]	7	58	7		13	1	5	77		
G9P[UT]		1	3				1			
G12P[6]	5	2	3		4					
G12P[8]		24	5							
Untypeable		5	1							
Myanmar^b^										
		1	2	3	4	5	6	7		
G1P[6]		15	7	2	30		31	3		
G1P[8]		3			2					
G2P[4]		1	1	14	9	22	3	1		
G2P[6]						2	2			
G2P[8]								1		
G3P[8]		5								
G9P[4]					1	1	2			
G9P[8]					7	1	20	20		
G12P[6]		6	50	103	45	1				
G12P[8]		3	7	8	37	2				
Mixed		6	5	2	11			1		
Partially typed		4	4	7	26	1	14	10		
Untypeable			7	1	5		2	3		
Lao PDR										
G1P[4]		6					1	1		
G1P[8]		53	32	47	15	96	14	145		
G2P[4]		66	44	2	6	57	76	3		
G2P[8]		2	1			1				
G3P[4]			2							
G3P[8]		7	32	80	92	7				
G3P[9]				1						
G4P[4]		1								
G4P[6]				1						
G8P[8]								1		
G9P[4]			1							
G9P[8]			40			6		1		
G10P[4]							1			
G12P[6]					1					
Mixed		19	2			1	2			
Untypeable		11	1							
Philippines										
G1P[8]						232	543	417	587	
G2P[4]						55	101	400	187	
G9P[8]						19	51	43	360	
Untypeable						8	22	7	133	
Mixed						3	7	28	13	
Unusual						5		28	40	
G1P[6], G2P[6], G1P[9]								9	13	
Vietnam										
G1P[4]					12	12	9			
G1P[8]					934	985	391	125		
G2P[4]					58	108	306	234		
G3P[8]					47	24	68	50		
G8P[8]							9	259		
Mixed					23	12	34	58		
Untypeable					93	48	26	92		
Others						12	9	17		
Malaysia										
	**2008–2010**									
G1P[8]	206									
G2P[4]	19									
G9P[8]	16									
G12P[8], G3P[9], G9P[9], G3P[8]	10									
Singapore^a^										
	**2005–2008**									
G1P[4]	4									
G1P[8]	125									
G1P[11]	1									
G2P[4]	49									
G2P[8]	5									
G3P[4]	1									
G3P[8]	61									
G9P[4]	5									
G4P[8]	1									
G9P[8]	68									

^**a**^In Singapore, the available data was between 2005 to 2008

^b^Myanmar: Surveillance data were collected during the intervals defined as 1. January – June 2009; 2. July 2009 – June 2010; 3. July 2010 – June 2011; 4. July 2011 – June 2012; 5. July 2012 – June 2013; 6. July 2013 – June 2014; 7. July – December 2014

Differences in the predominance of RV genotypes and newly emerging strains were identified over the surveillance period in Cambodia. The G1P[8] genotype was predominant in 2010, 2011, and 2012 (74, 66, and 79%, respectively), whereas genotype G2P[4] predominated between 2013 (54%) and 2014 (44%). The previously uncommon strain G8P[8] also emerged in 2014 (21%). The proportion of G8P[8] genotype detections increased further in 2015 (41%), in conjunction with the emergence of G9P[8] (10%). By 2016, the detection of genotype G9P[8] had increased to 18%, and G3P[8] became the most prevalent genotype (responsible for 63% of detections) [47].

In Indonesia, the surveillance results demonstrated a changing trend for the most prevalent genotype, from G1P[8] in 2009–2013 to G3P[8] in 2014–2018. From 2009– to 2013, G1P[8] was the most prevalent genotype circulating, which accounted for 38, 73.7, 67.5, 85.7, and 60.1% each year, respectively. G3P[8] became the most predominant strain in 2013, and this continued to 2015, accounting for 49,7, 82.5%, and 84,4%, respectively [49, 53, 55–57]. Another study reported that G3P[8]/[6] was also the predominant strain during 2015–2018 [58].

During the 7-year surveillance period in Lao PDR, the most predominant genotypes identified by year were G2P[4] (40%) and G1P[8] (32%) in 2009; G2P[4] (28%) and G9P[8] (26%) in 2010; G3P[8] (61%) and G1P[8] (36%) in 2011; G3P[8] (81%) in 2012; G1P[8] (57%) and G2P[4] (34%) in 2013; G2P[4] (81%) in 2014; and G1P[8] (96%) in 2015 [59].

From 2008 to 2010, the most common genotype in Malaysia was G1P[8] (82%). Other genotypes identified were G2P[4] (7.6%) and G9P[8] (6.3%). Approximately 4% of the samples were either mixed or untypeable (G12P[8], G3P[9], G9P[9], G3P[8]) [60]. A 2006 preliminary report in Sabah State showed that approximately 33% of samples were positive for RV, of which 33% were of genotype G4P [9, 84].

In Myanmar, the most common strains in 2009 were G1P [8] (28.3%) and G12P [8] (28.3%). G12P [8] was detected from 2009 to 2012, ranging from 28.3% in 2009 to 70% in 2011. G2P[4] became the most predominant strain in 2012–2013, followed by G1P[8] in 2013–2014. G9P[8] comprised only 1% of the RV strains in 2011 and increased to 97.5% in 2014. While in 2015, the majority (90%) of RV strains comprised G9P[8] (54%) and G3P[8] (36%). G9P[8] emerged in Myanmar in 2011 and was the most common strain in 2014 and 2015 [61, 85].

In the Philippines, 1949 (98.5%) RVA-positive stool specimens were successfully typed. The most common genotypes identified were G1P[8] (60.3%), G2P[4] (28.1%), and G9P[8] (5.7%). The frequencies of RVGE cases due to G1P[8] were similar in 2013 (72.0%) and 2014 (75.1%), but it decreased to 44.7% in 2015. Likewise, the frequencies of cases due to G2P[4] were similar in 2013 (17.1%) and 2014 (14.1%) but increased to 42.9% in 2015. The proportion of RVGE cases caused by G9P[8] did not change appreciably from 2013 (5.9%) to 2014 (6.9%) or 2015 (4.6%). Mixed genotypes, unusual strains, and animal strains were detected in specimens from 38 (1.9%), 22 (1.1%), and 10 (0.5%) children with RVGE,. Rare and unusual genotype combinations identified include three G1P[4], eight G2P[8], seven G8P [6], two G8P[8], and two G9P[4] strains. Also, animal strains were detected in specimens from 10 children, including 1 G3P[9] feline-like and nine G4P[6] porcine-like strains [63].

The predominant strain observed in Singapore was G1P[8] (18,3%), while G9P[8] (9,9%) was the second most common type observed among children in Singapore [65, 66].

In Thailand, G2P[4] was the most common genotype in 2008 (53,5%). G1P[8] was the predominant genotype in 2009, 2010, 2012, 2013, and 2014, accounting for 47.3, 35.8, and 63.9%, 60.4, and 56.2%, respectively. In 2011 (68.7%), 2015 (47.3%), and 2016 (89.8%), most RV strains were G3P[8]. Uncommon genotypes found were G1P7[5], G5P[19], G9P[4], and G9P[6], adding to the existing list of uncommon genotypes reported to circulate in Thailand [67–71, 73–76].

Based on RV diarrhea data from four sentinel hospitals in Vietnam from 2012 to 2015, G1P[8] was the most prevalent strain during 2012 and 2013, accounting for 80 and 82% of total genotyped samples, respectively. G2P[4] was found in 5% of samples in 2012 and 9% in 2013. In 2014 and 2015, the proportion of RVGE caused by G2P[4] increased to 36 and 28%, respectively. G8P[8] was not detected in 2012 and 2013, it accounted for only 1% of specimens in 2014, and it became predominant (31%) in 2015 [78].

### RV seasonality

Meteorological conditions have an indirect yet important impact on the epidemiology of human rotavirus infection. Weather-related low indoor relative humidity and indoor crowding may be key factors in the epidemiology of rotavirus disease. Hospitalizations for rotavirus gastroenteritis tended to be more common after a cold or dry month than after a warm or wet corresponding calendar month [86].

**Fig. 3:**
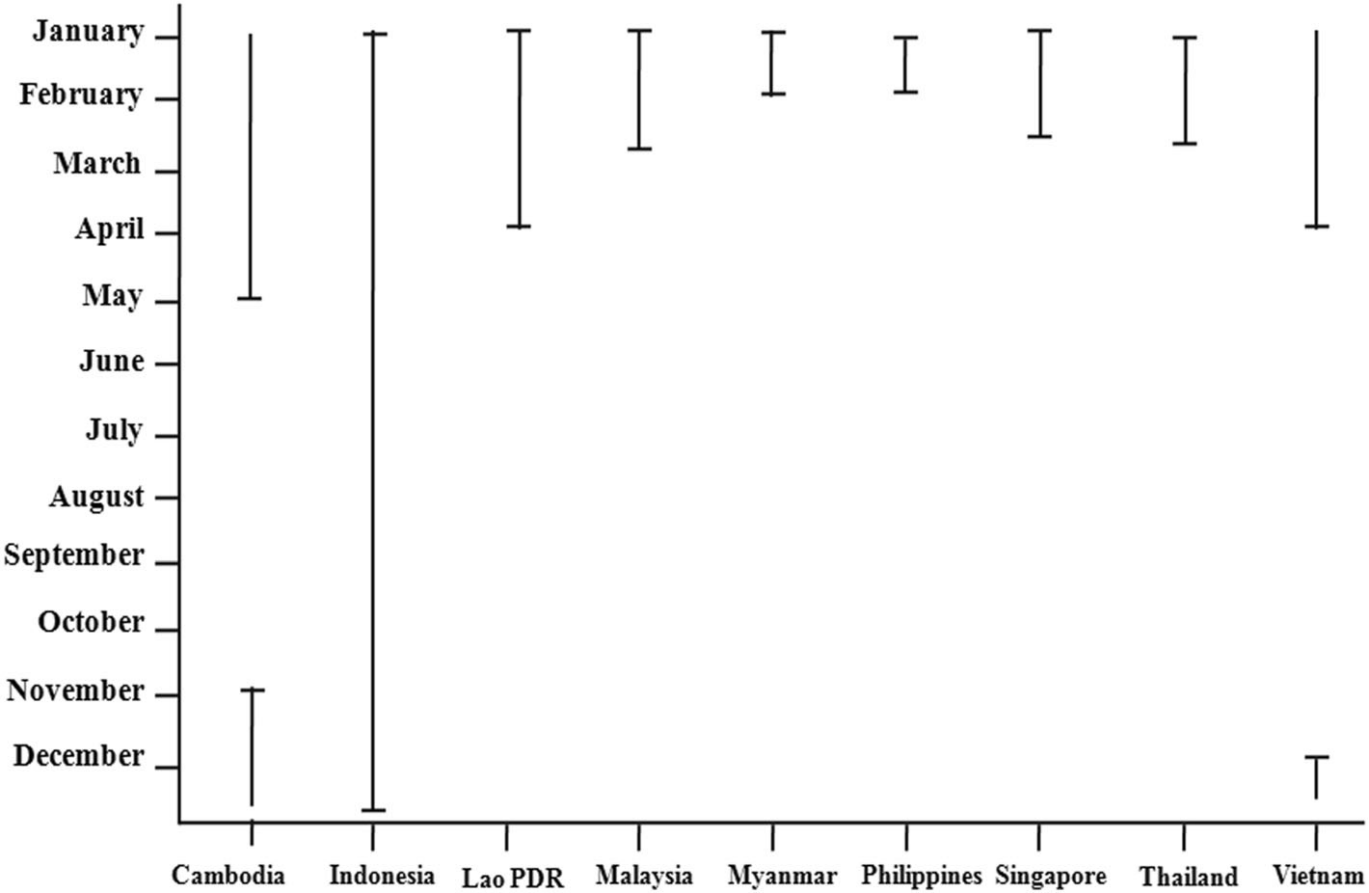
Seasonality of rotavirus in Southeast Asian countries

The temporal trend of Cambodian RV infection shows substantial year-round transmission with prominent peaks during colder, dry months. Peaks typically occurred between November and May [47]. In Indonesia, RV infection was present throughout the year and did not demonstrate clear annual seasonality [55]. Conversely, infection generally peaks during the rainy season in Singapore and Malaysia. An outbreak of RV infection was observed from January to March [65, 87]. Positive RV cases increased in number and proportion during the dry season (January–April) each year in Lao PDR [88]. In Myanmar and the Philippines, RV infection has a strong seasonal peak in colder, drier months, as seen in other Asian countries. The highest rate of RV infection occurred in January and February [61, 63]. RV cases in Thailand were most prevalent during the cooler months, specifically from January to March, but RV was detected every month in the northern part of Thailand, where the weather is relatively cooler compared to the rest of the country [68]. In Vietnam, RV was detected every month, but most RV gastroenteritis (GE) cases occurred between December and May [78]. Figure 3 shows the RV seasonality in Southeast Asian countries.

The seasonal pattern in RV varies by climatic region and is also associated with local weather. A reduction in RV rates was associated with increased temperature and precipitation [89]. There is a significant association between increased numbers of estimated positive RV cases and lower humidity, rain, and temperature. In children younger than 2 years old, RV was the pathogen most frequently identified in the winter, dry, or cool/dry seasons [90]. In tropical climates, the higher temperature was associated with a greater decrease in RV than in humid mid-latitude climates [91]. It had been suggested that the seasonal pattern may be driven by airborne transmission of the disease [92].

### RV vaccination

One of the best strategies for decreasing the global burden of disease is the development and implementation of effective vaccines [43]. RV is the most common cause of vaccine-preventable severe diarrhea [93]. The World Health Organization (WHO) recommended that RV vaccines be included in immunization programs in the European region and the Americas in 2006. In 2009, following efficacy studies in low-income countries (LICs) and lower-middle-income countries (LMICs) in Africa and Asia, the WHO recommended the use of RV vaccines in all National Immunization Programs (NIPs) [94]. RV vaccines had been introduced in 101 countries by the end of 2018, and global coverage was estimated to be at 35% [95]. Most countries in Southeast Asia have not yet introduced national RV vaccination programs. Among Asian countries, only the Philippines, and recently Thailand have introduced the vaccines on a limited basis [96].

The Global Alliance for Vaccines and Immunizations (GAVI), also known as the Vaccine Alliance, actively supports RV vaccination by subsidizing the cost in eligible countries. In LMIC, the budget impact is an important criterion for funding new interventions, particularly for large public health investments such as new vaccines. By the end of 2018, GAVI had funded RV vaccine introductions in 45 countries [97].

### RV vaccine history

Research to develop a safe, effective RV vaccine began in the mid-1970s when investigators demonstrated that previous infection with animal RV strains protected laboratory animals from experimental infection with human RVs [98]. The first multivalent live oral reassortant vaccine developed was RotaShield® (a rhesus RV tetravalent [RRV-TV] vaccine) in the late 1980s [99]. The RRV-TV vaccine was licensed in August 1998 for routine use in children in the United States at 2, 4, and 6 months of age due to its proven efficacy [100]. In 1999, RotaShield® was voluntarily withdrawn from the U.S. market due to an increased risk of intussusception within 3 to 14 days after the first dose in infants < 3 months of age [101].

Due to the past association of intussusception and the earlier RV vaccine, large safety studies were performed both for RV1 and RV5 before market authorization [102]. Rotarix® vaccine were highly efficacious in protecting infants against severe rotavirus gastroenteritis [66, 103] and were not associated with an increased risk of intussusception [104–106]. The pentavalent rotavirus vaccine (RotaTeq®) was highly efficacious against severe rotavirus gastroenteritis and provided substantial protection against rotavirus gastroenteritis of any severity. A significantly increased risk of intussusception in vaccine recipients was not detected [107]. In February 2014, WHO reviewed global intussusception data and found that the risk of intussusception following current rotavirus vaccines remains small compared to the benefits of preventing the impact of severe diarrhea [108].

At the end of 2018 there are four globally available WHO-prequalified oral vaccines (Rotarix® and RotaTeq®, Rotavac® and Rotasiil®) [109], one rotavirus vaccine licensed in China (Lanzhou lamb RV vaccine), one in Vietnam (Rotavin-M1), and there are several candidates in development [110].

Two RV vaccines, Rotarix® and RotaTeq®, have been developed by Glaxo Smith Kline and Merck, respectively. Rotarix® is a live attenuated monovalent vaccine derived from the most common human RV strain, G1P[8]. RotaTeq® is a live attenuated pentavalent vaccine containing mono-reassortant strains with genes encoding the human G1, G2, G3, G4, and P[8] protein in the genetic background of a bovine RV strains [39, 100]. These vaccines are highly effective for the global prevention of severe diarrhea and are included in the NIPs or phased subnational introductions in 101 countries by the end of 2018 [95, 111].

Rotavac® (Bharat Biotech International Limited) is a monovalent human-bovine RV vaccine. The vaccine consists of the 116E RV strain, which is a naturally occurring reassortant strain G9P[11], containing 1 bovine RV gene P[11] and 10 human RV genes [112]. Rotavac® is the first to be introduced into a public vaccination program as of April 2016 when it was introduced in four states in India [113]. Rotasiil® is a live attenuated human-bovine reassortant pentavalent RV vaccine that contains genes encoding the VP7 of serotypes G1, G2, G3, G4, and G9. In March and September 2018 Rotavac® and Rotasiil®, respectively achieved WHO prequalification. Rotavac® has a vaccine efficacy of 53.6% for severe RV diarrhea in India [112], while Rotasiil® has efficacies of 60.5 to 66.7% in India [114] and Niger respectively [115]. Rotasiil® can safely be delivered with decreased dependence on the availability of a cold chain [116].

The Lanzhou Institute of Biological Products manufactures the Lanzhou Lamb RV vaccine (LLR). It is a monovalent lamb vaccine strain G10P[12], attenuated by cell passage [117], and was licensed in China in 2000. When given to children between 9 and 35 months old, one dose of the LLR vaccine conferred partial protection [118]. Vaccine effectiveness in children under 5 years of age was recently estimated at 35% (13 to 52%) against RV diarrhea and 53% (15 to 75%) against moderate-to-severe RV diarrhea based on a large case-controlled study [119].

Rotavin-M1 vaccine is manufactured by the Center for Research and Production of Vaccines and Biologicals and was licensed for use in Vietnam in 2012. The vaccine was derived from an attenuated strain, G1P[8], isolated from a Vietnamese child. A clinical trial found the vaccine to be safe and immunogenic in Vietnamese infants [120].

Another candidate RV vaccine, RV3-BB, was developed from a neonatal strain G3P[6] identified in Australia, with ongoing early clinical studies conducted in New Zealand and now underway in Indonesia. It was also successfully implemented for vaccination of neonates [121]. Table 4 describes the comparison of all oral RV vaccines developed and used so far.

**Table 4:** Comparison of oral rotavirus vaccines

Rotavirus Vaccines	Rotarix® (GSK) [100]	RotaTeq® (Merck) [100]	Rotavac® (Bharat Biotech) [112]	RotaSIIL® (Serum) [116]	Rotavin (Polyvac) [120]	LLR (Lanzhou) [118, 119]	RV3-BB (Bio Farma) [121]
**Licensure**	Several countries, 2006	Several countries, 2006	India, 2014	India, 2017	Vietnam, 2012	China, 2000	Clinical trial phase IIb
**Prequalification**	Yes	Yes	Yes	Yes	No	No	No
**Strains**	Monovalent, human derived G1P[8]	Pentavalent, WC3 G6P[5] bovine reassortant G1–4,P8	Monovalent, human neonatal derived G9P[11]	Pentavalent, UK Bovine G6P[5], reassortant G1–4, G9	Monovalent, human G1P[8]	Monovalent, Lamb G10P[12]	Monovalent, human neonatal G3P[6]
**Number of doses**	2	3	3	3	2	3	3
**Age, first dose**	6 weeks	6 weeks	6 weeks	6 weeks	6 weeks	2 months	New born: 0–5 days Infant: 8 weeks
**Age, last dose**	10 weeks	14 weeks	14 weeks	14 weeks	14 weeks	36 months	New born: 14 week Infant: 18 weeks
**Dosage**	10^6^ median CCID_50_ of live attenuated human G1P[8] RV	2.0–2.8 × 10^6^ infectious units per reassortant	10^5^ fluorescent focus unit (FFU) of live rotavirus	10^5.6^ infectious units per reassortant	10^6.3^ FFU/dose of live attenuated human G1P[8] particles	> 5.5 log CCID_50_	8.3–8.7 × 10^6^ FCFU/ml
**UNICEF price per course for GAVI-supported countries, 2020** [122]	$4.58	$9.60 (RotaTeq is no longer an option available to GAVI-supported countries)	$2.55	$4.65 (1-dose vial) $2.85 (2-dose vial)	$17.60	$72 for the three-dose series	

Intussusception, neutralizing antibodies present in breast milk, as well as the lower vaccine effectiveness in less developed settings has stimulated interest in an alternative, parenteral approach to immunization [123–126]. The inactivated rotavirus particles, protein sub-units or virus-like particles (VLPs, structurally-similar to live virus) are being investigated as rotavirus vaccine candidates [125, 127, 128].

Three types of animal models have been used to evaluate protective efficacy of VLPs: infection models (adult mice and rabbits), disease models (gnotobiotic piglets), and models evaluating passive protection (neonatal mice and calves) [129]. Gnotobiotic pig was used to assessed the immunogenicity and protection of a candidate inactivated rotavirus vaccine (IRV), the human strain CDC-9 (G1P[8] [130] and attenuated Wa human rotavirus [AttHRV] or non replicating Wa 2/6 rotavirus-like particles [131]. Mice, rabbits, and piglets were used to evaluate the efficacy of VPL such as 2/6-VLPs (consisting of VP2 and VP6) [129] and RF 8–2/6/7-VLPs [132]. Human clinical trial recently assessed in South African toddlers and infants was done for the subunit vaccine P2-VP8-P[8] [133, 134].

### Vaccine efficacy

RV vaccination does not completely protect young children against infection, but it reduce the severity of RVGE [135]. RV vaccines are highly effective in preventing severe gastroenteritis in young children during the first 5 years of their life, particularly in developed countries [136]. The SES of a country seems to influence RV vaccine effectiveness [137]. Vaccination was predicted to prevent 93, 86, and 51% of severe RVGE in high, middle, and low SES, respectively [126]. Analysis of the data for the Asia region found median vaccine effectiveness of 94% in low child mortality countries, 64% in medium child mortality countries, and 49% in high child mortality countries [138]. Factors that might contribute to this phenomenon including gut microbiota, genetic factors, transplacental antibodies, enteric pathogens, and environmental enteropathy [139, 140]. Evidence suggests that vaccine efficacy may vary by setting, due to regional differences in circulating RV vaccine strains and reduced efficacy of oral vaccines in settings with a high prevalence of malnutrition and gastrointestinal infections [141]. Pooled efficacy estimate of Rotarix® and RotaTeq® against severe RVGE in industrialized countries is 88% during the first year of age and 83% during the second year. However, RV vaccine efficacy is much lower in countries where the mortality rate for children under 5 years of age is high [142]. The efficacy of Rotarix® and RotaTeq® in the U.S. depends on the level of exposure during the RV season [143]. It can be concluded that vaccine efficacy is affected by individual factors such as nutritional level, gut microbiota, genetic factor, transplacental antibody and environmental enteropathy and external factors including SES, circulating vaccine strain, childhood mortality rate, and RV season in each country.

Additionally, RV vaccination confers herd protection among infants and children under 5 years old who had not been vaccinated [144–146]. In developing countries with lower RV vaccine efficacy and coverage, indirect protection gain from herd immunity is more significant than in industrialized countries where vaccine efficacy and coverage exceed 90% [142].

### Vaccine introduction in Southeast Asia

Many countries in Southeast Asia have not implemented national RV vaccination programs including Cambodia, Indonesia, Lao PDR, Malaysia, Myanmar, Singapore, Thailand and Vietnam. One reason is because of uncertainties regarding the cost-effectiveness of incorporating RV vaccination into the NIP. In addition, the vaccine’s decreased efficacy in LIC settings has discouraged its introduction. Prevention of diarrhea in these countries has focused on patient treatment and the management of water quality, sanitation, and hygiene [84].

In Indonesia, the RV vaccine has been commercially available since 2011. Indonesia’s national vaccine manufacturer, PT. Bio Farma, Bandung, is developing an RV vaccine using G3P[6] strain in collaboration with the Murdoch Children’s Research Institute in Melbourne, Australia [125]. Bio Farma is currently driving clinical development, intending to introduce the vaccine into the Indonesian NIP by 2021, and eventually develop a product for the global market [128].

Currently Rotarix® and RotaTeq® are commercially available in Malaysia through private health providers [147]. However, RV vaccine is not included in Malaysia NIP because it is not considered permissible under Islamic shariah law (halal) [84]. The current oral rotavirus vaccines use porcine trypsin in the manufacturing process [148]. There are also concerns about competing public health priorities and price [84]. The Health Ministry recently said the RV vaccine would be included in NIP if the associated mortality rate for children aged 5 and below exceeded 10%. However, the childhood mortality rates in Malaysia was 0.5% in 2014 and 2.9% in 2015 [149].

In Myanmar and Lao PDR where individual incomes are relatively low, international assistance will support for the RV vaccine introduction in 2020. The total amount of GAVI support for Myanmar is $4,088,000 [150]. Lao PDR is also planning RV vaccine introductions into the NIP in near future and assistance will be provided the Asian Development Bank and GAVI through an accelerated transition program [84].

In July 2012, the Philippines became the first Asian country to introduce RV vaccines into its NIP. The Philippines has initially focus on immunizing children living in the poorest communities, which have the highest child morbidity and mortality rates from the diarrheal disease [151]. The target population was identified by the Department of Social Welfare and Development, but there were challenges with nationwide vaccine distribution. In 2014, vaccine introduction was limited to the Caraga region, where it was co-administered with oral polio vaccine and the pentavalent vaccine. By 2015, vaccine coverage was close to 90% in the province of Agusan del Sur within this region but subsequently decreased due to a supply shortage [84]. In Agusan del Sur, the RV vaccine became available to the poorest quintile in September 2012; in January 2013, availability was expanded to all age-eligible children in two municipalities, San Francisco and Prosperidad; it was available to the entire province in July 2014. RV vaccine introduction was associated with a substantial decline in diarrheal hospitalizations and outpatient consultations for diarrhea in Agusan del Sur, Philippines [152].

Two live-attenuated, orally administrable RV vaccines Rotarix® and RotaTeq® were licensed in Singapore in October 2005 and July 2007, respectively [106]. To date, RV vaccination is optional in Singapore [65].

The National Vaccine Committee of Thailand considered the introduction of an RV vaccine in 2010 in Sukhothai and Petchabun. Sukhothai province began a routine immunization program with an RV vaccine in October 2011. Evaluation of the first introduction was completed in 2017 and concluded that RV vaccine was highly effective in preventing diarrheal hospitalizations and conferring herd protection among older children who had not been vaccinated [144]. Therefore, universal RV vaccination for infants has been implemented in Thailand since January 2020.

Vietnam is located in a region of high RV infection incidence and eligible for financial support to introduce vaccines into the expanded program of immunization (EPI) from the GAVI [153]. In 2012, the local vaccine manufacturer Polyvac licensed Rotavin-M1, which is based on an attenuated G1P[8] strain. Rotavin-M1 will be offered to children less than 1 year through a two-dose schedule, vaccinating infants at 2 and 4 months [120]. Rotarix® and RotaTeq® are also licensed in Vietnam and are available in the private sector, with approximately 590,000 doses imported since 2017. That same year, the Government approved the introduction of RV vaccination into Vietnam’s NIP by September 2019 with GAVI support. In 2021, the national government will pay for 80% of the vaccine cost, while GAVI will cover the remaining 20% and all operational costs. By 2022, all costs will be covered by the government [84].

### Health and economic impacts of RV vaccination

In LMICs, understanding the short- and long-term impact of intervention adoption on national budgets is critical for ensuring program sustainability [154]. Both budget impact and cost effectiveness are key criteria, among others, for policy makers deciding how to allocate limited resources [155].. Vaccination would be considered a worthwhile investment for improving general childhood development and health levels in most LIC. The highest reduction in burden would be achieved in countries with a high disease burden (≥200 RV deaths per 100,000 children under 5 years old), but a similar reduction would be achieved in countries with a medium burden (100–200 RV deaths per 100,000 children under 5 years old) because disease burden reduction also depends heavily on population size and country-specific vaccine efficacy adjusted for local RV serotype distributions [156].

For GAVI non-eligible countries, the price for Rotarix® is $2.49–7.27, and for RotaTeq® is $3.65–5.09 [157]. For GAVI-eligible countries, the price per dose will depend on the country’s gross national income per capita averaged over the previous 3 years. As such Indonesia, Malaysia, the Philippines, Singapore, and Thailand are fully not eligible for GAVI vaccine prices and will have to rely on self-financing [158]. For LIC in Asia, introducing vaccines would halve RV-related deaths and medical visits, leading to significant cost reductions [159].

**Fig. 4:**
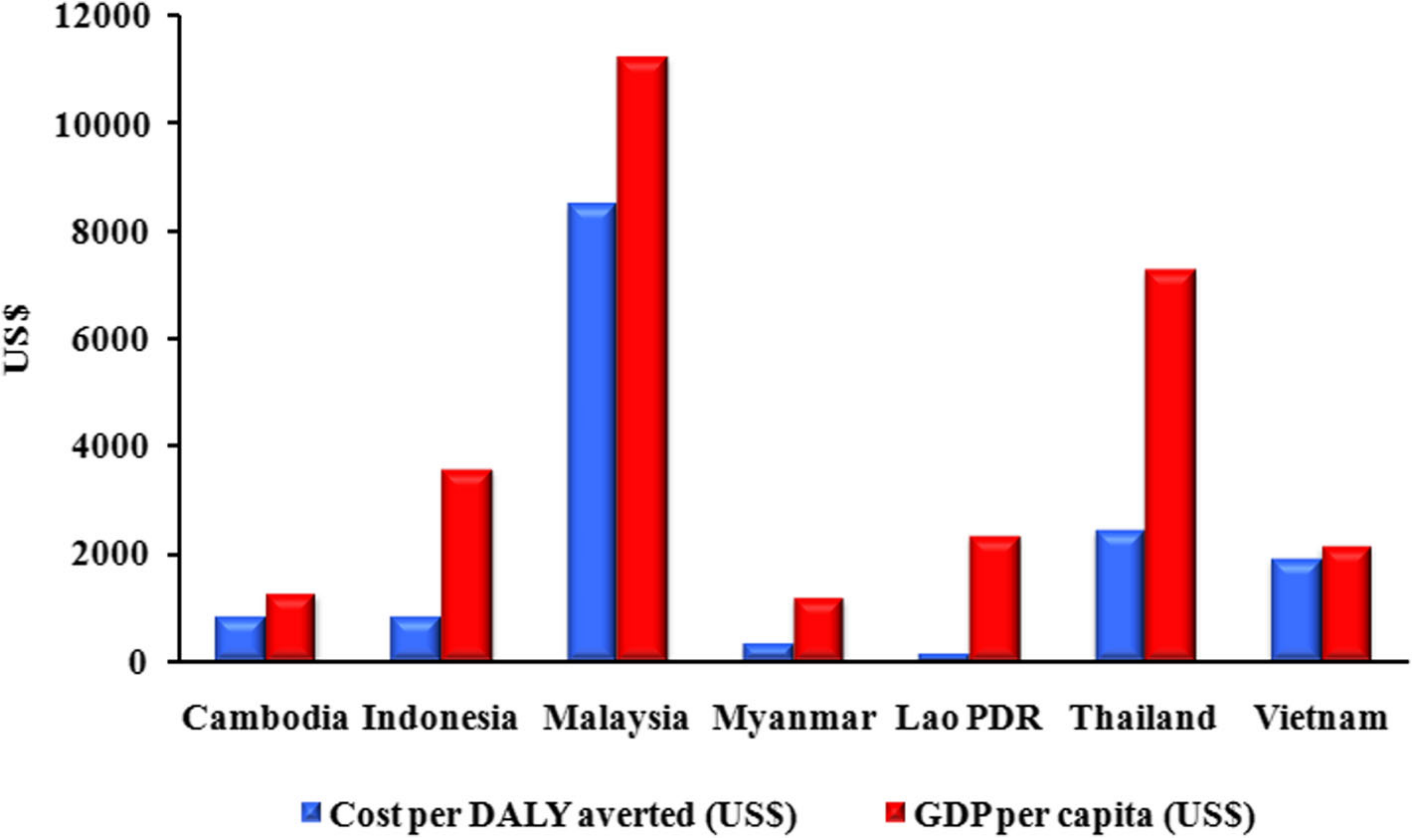
Comparison between cost per DALY averted and GDP per capita in Southeast Asia. Introducing the rotavirus vaccine in Southeast Asia is highly cost-effective because the ratio is less than one [79, 107, 137, 161]

WHO-CHOICE (CHOosing Interventions that are Cost-Effective) uses the GDP as an indicator to develop the following widely referenced categories of cost-effectiveness. Disability-adjusted life years (DALYs) averted is a widely used indicator that allows easy comparison with a ‘no vaccination’ strategy and with others public health interventions. The ratio between costs per DALY averted and GDP per capita less than one is defined as highly cost effective. The ratio between 1 to < 3 and ≥ 3 are defined as cost effective and not cost effective, respectively [160]. The lowest and highest GDP values were in Myanmar with $1326 and Malaysia with $11,239, respectively [79]. Figure 4 shows the comparison between costs per DALY averted with each country’s GDP in 2018. We excluded the Philippines because the RV vaccine already included in the country’s NIP and Singapore because Singapore is a high-income country. According to categories of cost-effectiveness, RV vaccine introduction into Southeast Asia countries is highly cost-effective because the ratio between costs per DALY averted and GDP per capita it is less than one.

## Conclusions

According to 2008–2018 RV surveillance data for Southeast Asia, 40.78% of all diarrheal cases were causes by RV. Acute diarrheal disease caused by RV is still a major cause of morbidity and mortality in children under 5 years old in Southeast Asia. Among all assessed countries, the most predominant genotype distribution of RV changed from G1P[8] and G2P[4] into the rare and unusual genotypes G3P[8], G8P[8], and G9P[8]. Although the predominant RV strain has been changed, but the seasonality of RV infection remains unchanged. Continuous surveillance is necessary to determine whether they are regional genotype differences. Epidemiological data on RV prevalence will greatly facilitate vaccine development. In the mean time, the development of new vaccines will be needed if RVs are able to evade current vaccine immunity. More effective vaccines may also further decrease RV infection in children in LIC and LMIC, where currently available vaccines provide moderate efficacy. RV vaccine efficacy is affected by individual factors and external factors. Although most countries in Southeast Asia have not yet introduced national RV vaccination programs, such introduction is projected to be highly cost-effective because the ratio between costs per DALY averted and GDP per capita it is less than one.

## Data Availability

Not applicable.

## Abbreviations

AGE: Acute gastroenteritis
DALY: Disability-adjusted life year
EM: Electron microscopy
G: Glycoprotein
GAVI: Global Alliance for Vaccine and Immunization
GDP: Gross domestic product
LIC: Low-income countries
LMIC: Low-middle income countries
NIP: National Immunization Program
NSP: Non-structural protein
P: Protease
RV: Rotavirus
RVGE: Rotavirus gastroenteritis
SES: Socioeconomic status
VP: Viral protein
WHO: World Health Organization
